# The Hospital. Nursing Section

**Published:** 1906-11-03

**Authors:** 


### The Hospital.

### Nursing Section.

Contributions for "THE HOSPITAL," should be addressed to the EDITOR, "THE HOSPITAL" NURSING SECTION, 28 & 29 Southampton Street, Strand, London, W.C.

No. 1,050.—VOL. XLI. SATURDAY, NOVEMBER 3, 1906.

### Notes on News from the Nursing World.

### OUR CLOTHING DISTRIBUTION.

IF contributions to our Clothing Distribution do not come in so rapidly as we could wish, applications in the interests of those who need relief do not tarry. How immensely appreciated these gifts of our readers are is always shown by the grateful letters sent to us from the matrons of hospitals and infirmaries after their reception. We hope that these communications will be more numerous than ever on the morrow of Christmas 1906, and that there will be a goodly list of acknowledgments from this week onwards. We have to acknowledge the receipt of contributions from Policy 4781, M. E. N., and Miss Webbe, London. All parcels must be sent addressed to the Editor, 28 and 29 Southampton Street, Strand, London, W.C., and should have "Clothing Distribution" written on the outside.

### MISS NIGHTINGALE, THE SOLDIER'S FRIEND.

AT a dinner given last week at Carlton Hall, Brixton, to commemorate the fifty-second anniversary of the charge of the Light Brigade at Balaclava, several survivors being present, the toast of "Miss Florence Nightingale, the Soldier's Friend," was given and aroused much enthusiasm. Mr. T. H. Roberts, who proposed it, said that, in reply to a telegram sent to her on behalf of the survivors, Miss Nightingale had wired, "Miss Nightingale sends cordial thanks and hearty good wishes to her old comrades." More applause followed the reading of the telegram, which evidently afforded the twenty-three "old comrades" of Miss Nightingale the greatest pleasure.

### MR. HALDANE LOOKING INTO NURSES' AMUSEMENTS.

ON Thursday last week Mr. Rendall asked the Secretary of State for War whether he could explain why nurses at Netley Hospital, and in the service of the Army generally, were not permitted, when off duty, except on furlough, to take part in public or private dances; and also "whether he would at once free the nurses from this interference with their liberty." Mr. Haldane, in reply, desired the hon. member to postpone his question for a week, as "he was looking personally into the amusements of these nurses." It is added that this announcement was received with laughter. A representative of the nurses at Netley has published the fact that the great body of the Army sisters there approve the regulation complained of.

### THE TRAINING OF ZULU GIRLS.

ON Thursday evening last week a very interesting meeting in connection with the Guy's Hospital Missionary Society was held in the Court-room of the hospital. The speaker was Miss Mallandaine, a former nurse, who finished her training in 1901, and has since worked in the mission field. She is now stationed at the Etalanen's Mission Station in Zululand, where she is hoping to start a hospital upon her return at the end of the year. Miss Mallandaine has a project in view for the training of Zulu girls as nurses, who would in their turn spread widely through the land the knowledge they had acquired, so that in time many of the native practices may be superseded by humane and skilful methods. A maternity ward for native women is to be opened, and eventually also one for Europeans. The institution is to be self-supporting in the future, but at present it will have to be maintained. Many curios were shown by Miss Mallandaine, who explained their uses. The audience was composed mainly of nurses, and a collection for the new mission hospital was made.

### THE DEAN OF MANCHESTER ON THE PERSONALITY OF A NURSE.

IN an address delivered last week to the nursing staff of Crumpsall Workhouse Hospital, Bishop Welldon, Dean of Manchester, having referred to the wonderful change which had come over the nursing profession during the last 50 years, went on to speak of the qualities required to make a good nurse. He expressed a hope that the Crumpsall nurses were allowed sufficient leisure for the recreation of mind and body. He could not conceive that "a lady who was not high in mind and soul," however great her skill might be, could be a first-rate nurse. Unless he altogether misunderstood the problem of healing, there was something in the personality of the nurse which counted when all other qualifications were exhausted.

### THE NURSE IN THE ELEMENTARY SCHOOLS.

IT is both interesting and satisfactory to hear of a member of the House of Commons spending his time in watching the work of the Queen's Nurses. Mr. Macnamara, M.P., in addressing a meeting of school teachers at Liverpool on the Education Bill, mentioned in the course of his address that he had occupied the day in some of the poorest schools in the city, and had carefully observed in these buildings the ministrations of the Victoria District Nurses. His conclusion was that the citizens of Liverpool ought to go on their knees and thank God for the beneficent labours of those good women. He added that he should feel happy if that work were carried on universally in our great cities. We may hope, therefore, that whatever influence he possesses as a member of the Legislature or otherwise, will be used in favour of the introduction of the nurse in the elementary school. She is often at least as much wanted there as the teacher of the three R's.

### NEW HOSPITAL FOR WOMEN.

The resignation by Miss Barling of the post of matron of the New Hospital for Women was handed in more than a fortnight ago; but the date of her departure is not yet settled, and depends upon the time her successor is appointed. She prefers not to give any reason for her resignation.

### NURSING SMALL-POX PATIENTS UNDER DIFFICULTIES.

The disadvantages of nursing small-pox patients in tents are described in an interesting manner by a contributor this week who went through the experience herself in a recent epidemic. Of course, a woman ought never under any circumstances to have been left alone in such an isolated spot at the mercy of delirious patients, or, worse still, at that of a frequently tipsy Irishman, who, even when sober, was not a desirable kind of person. We are glad to know that such a state of things in that part of the North of England is no longer possible. The tents were taken down at the close of the epidemic, and a wooden hospital has been erected in their place. Whilst the arrangements of some isolation hospitals are far from being all that could be desired, nurses are now at least provided with properly heated rooms and the attendance of one of their own sex.

### MIDWIVES AND TOTAL ABSTINENCE.

The report which we give on another page of the meeting of the Certificated Midwives' Total Abstinence League will be read with interest. It is clear that the movement, which owes much to Miss Alice Gregory and other ladies, has taken root; and we are glad that it should be so. Without subscribing to all the opinions which were expressed on Friday, or insisting upon the necessity of a qualified midwife being a total abstainer, we fully recognise that, apart from any moral issue, this may be a distinct advantage. As Miss Paget reminded her hearers, the cases which come before the Central Midwives Board involving penal consequences are largely those of women who have given way to intemperance. It does not follow that only a total abstainer can be a skilful midwife, but there is no doubt that any abuse of alcohol renders the most skilful of midwives unfit for her work. Of course, we know that the same remark applies to nurses generally and more or less to all workers. But the nature of a midwife's duties, the peril in which she may place the lives of both mother and child by indulgence in drink, constitutes a special need for self-control and sobriety.

### ENGAGEMENTS IN NURSING HOMES.

The experience of two correspondents in connection with nursing homes, which is given in another column, affords a further illustration of the folly of accepting an engagement at a nursing home without making the fullest inquiries. The need for the registration of nursing homes has been insisted upon by us repeatedly; but even now the exercise of ordinary forethought would do much to prevent trouble. A nurse should, if possible, see the Home which she proposes to enter, and she should always submit the agreement which she is expected to sign to a lawyer or a good business man before she appends her name to it. By complying with these two simple precautions considerable disappointment, if not actual suffering, might be avoided.

### DISCLAIMER BY THE JUBILEE INSTITUTE.

The Council of Queen Victoria's Jubilee Institute have been compelled to signify that there is no connection between the Institute and the Walthamstow, Wanstead, Chingford, and Highams Park Joint Nursing Association. The authorities of the latter, in sending out their report, printed as a portion of it a list of the Council of the Institute, and the impression was conveyed that the local organisation was acting in co-operation with it. The managers of the Association ought, of course, to know that unless they are affiliated to the Institute, they have no right to make use of its name.

### ROYAL VISIT TO THE RYDE COUNTY HOSPITAL.

On Thursday last week Princess Henry of Battenberg, who was accompanied by Lady Adela Cochrane and attended by Miss Minnie Cochrane, visited the Royal Isle of Wight County Hospital, Ryde, of which she is President. The Princess, who was received by Mr. T. B. Cochrane, Deputy-Governor of the Island; Mr. M. Maybrick, Vice-Chairman of the Committee; and Miss Antram, the matron, inspected the new out-patients' buildings now in course of erection, and afterwards each ward was visited in turn; and the gracious and sympathetic intercourse of her Royal Highness with the patients was a source of pleasure to all. The Princess was specially interested in the Queen Victoria Ward for children, and it was delightful to see her tenderness for some of the poor litle sufferers. After all the wards and several other parts of the hospital had been visited tea was served by Miss Antram in her private sitting-room. The Princess expressed satisfaction at all that she had seen during her visit. Shortly after 6 p.m. she left by motor for Osborne Cottage.

### THE ACTION AGAINST THE MATRON OF A MATERNITY HOME.

We have received an interesting letter from the Rev. Richard Free, of St. Clement's Vicarage, Fulham, in which he expresses his regret "at the apparent miscarriage of justice in the case recently heard at the Brompton County Court, Jukes *v.* Heatley." The fact that the Judge overruled the definition of a trained nurse which the matron of the Home herself imposed seems to establish, in Mr. Free's opinion, a precedent that may have far-reaching effects. He adds: "The Judge's decision in this case practically says that an applicant for training may promise adherence to rules which she never intends to fulfil, she may refuse to pay the full fees required of her, and then, because the training is not up to her requirements, she may demand her money back." We are afraid that unless Miss Heatley cares to run the risk of incurring the serious expense of an appeal against the County Court judgment, nothing further can be done in the matter.

### QUEEN'S NURSES AND SLEEPING ACCOMMODATION.

The Queen's Nurses at Hull will, in a short time, be provided with ample sleeping accommodation. The original house in Charlotte Street is quite inadequate for the needs of the staff, and the enlargement which has been rendered possible by the liberality of Sir James Reckitt, the President of the Association, will enable each nurse to have a room of her own. This is even more necessary in the case of a Queen's nurse than in that of a probationer in a hospital, and we are glad that in future none of the staff attached to the branch of the Jubilee Institute in Hull will be required to put up with cubicles. But why did the Committee at Hull engage nurses whom they could not adequately house?

### MEMORIAL TO A MATRON.

At the annual meeting of the Western Eye Infirmary, held in Exeter on Friday last, the President read the report, the first portion of which had reference to the recent death of Miss Georgina Kinninmont, to which we alluded at the time of the event in September. In the report there was a passage to the effect that Miss Kinninmont "had occupied the position of matron for the past sixteen years, and had during that period given all her well-known energy, aid and ability to the welfare of the institution, which, to a great extent, owes its present high state of efficiency to her untiring and self-sacrificing efforts." Subsequently, the Chairman having alluded to Miss Kinninmont as "the best friend the hospital ever had," Dr. Gordon raised the question of a memorial to her. He observed that Miss Kinninmont had directly, and indirectly, increased the funds of the hospital, and had been the means of getting the new wing. "How she managed to do her work, as not merely the matron, but also as house surgeon and house-governor, was a miracle to those who watched." He proceeded to say that the Committee had carefully considered a proposal to commemorate her services. They had come to the conclusion that the best possible manner of commemorating her name and the devotion she showed, and of doing it in the way she would herself have wished, was to call the children's ward the Kinninmont Ward, and to place in the entrance hall of the hospital a tablet setting forth her services. This form of memorial found favour with the meeting, and it was unanimously agreed to. Probably none more acceptable to the friends of the late esteemed matron could have been adopted.

### A HOLIDAY MEETING OF THE SOCIAL UNION.

The Bristol Branch of the Nurses' Social Union had an enjoyable, though rather novel, meeting the other day. Their hostesses, Mrs. Barnes and Miss Fry, rightly thought that town workers would appreciate an afternoon in the country, and accordingly the meeting was held at Portishead on a day of glorious autumn sunshine. The lecturer was unavoidably prevented from coming at the last moment, but the nurses enjoyed nature studies in the shape of kittens, puppies, calves, chickens, and pigs, and geese to watch and feed; blackberrying and tea in an orchard completed an ideal afternoon of restful enjoyment, while the "loan collection" in the background lent the touch of instruction the occasion demanded. The Union aims at elasticity of method, and the character of the meetings is left largely in the hands of the local organisers to determine. An occasional "holiday" meeting can but be helpful.

### MASSAGE CLASSES AT A DUBLIN HOSPITAL.

The Massage Classes which have been given during the past year at Dr. Steevens' Hospital, Dublin, have been well attended, and certificates successfully gained by seven members. The Swedish system has been taught by Miss Studley, Principal of the Irish School of Massage, and Dr. Toard, who presented the certificates, emphasised the value of massage, and recommended every nurse who was able to qualify herself in this branch. He also congratulated the matron on the massage class as being one of her many successful efforts for the benefit of the hospital. Later in the evening an excellent programme of music was rendered at the Nurses' Home, and refreshments were served in the library.

### SHORT ITEMS.

"The History of Nursing in the British Empire" has been written by Mrs. Sarah A. Tooley, author of the "Life of Florence Nightingale," and will be published almost immediately by Messrs. Bousfield and Co., 12 Portugal Street.—Between the 23rd and 26th October a series of well-attended meetings of nurses in Yorkshire on the subject of the Royal National Pension Fund have been addressed by the secretary, Mr. Louis H. M. Dick. The first gathering took place at the York County Hospital, and amongst those present were Miss Tuke, the matron, and Miss Morgan. This was followed by meetings at the Leeds Union Infirmary; the Royal Infirmary, Huddersfield; the Royal Infirmary, Bradford; and the Fever Hospital, Seacroft, Leeds. Amongst those who attended the various meetings were the matron of the Leeds Union Infirmary, Miss Deakin, of the Women and Children's Hospital, Leeds; Miss Puxley, matron of the Royal Infirmary, Halifax; Miss Kidson, matron of St. Luke's Hospital, Halifax; and Miss Hodges, matron of the Bradford Royal Infirmary.—The dates of the three lectures to certified midwives at the Midwives Institute, 12 Buckingham Street, Strand, announced for Tuesdays in November have been unavoidably changed to Friday, 16th; Tuesday, 20th; and Friday, 30th. Dr. Fairbairn will be the lecturer instead of Dr. Calder, who, owing to unexpected engagements, has been obliged to cancel previous arrangements.

### The Nursing Outlook.

"From magnanimity, all fears above;
From nobler recompense, above applause,
Which owes to man's short outlook all its charm."

### THE SPIRIT IN THE WORK.

Life in a public institution, where the administration is of the best and the necessary discipline is enforced with absolute justice, has something in common with life in a marching regiment. Human nature being what it is men and women cheerfully move in crowds in directions which they, as individuals, would never take. In a battle, when the order to advance is given, the spirit which animates each individual unit of a regiment represents the aggregate spirit of the whole number, because of the sympathy which is engendered by the association of a number of men together in pursuit of the same object. So in a great public institution every individual member of the nursing staff must acquire strength from the spirit which she may gain by association with her sisters day by day. It is therefore of the first importance to the success of all administrators that the individual workers shall be under a benevolent discipline, which knits them together and causes them to be animated by the same high spirit of efficiency, whilst discharging the duties which each day's work involves. An ordinary observer, who has any knowledge of the inner working of an institution, must be struck with the difference which sometimes characterises the appearance and attitude of the staff in a large, as compared with a small institution. There need be no such difference providing the chief official is imbued with the right spirit, and can so animate each and all of those who work under his or her direction. Hence the presence or absence of *esprit de corps* means efficiency or failure in fact.

In the nursing body the public very often complain with justice of the conduct of certain nurses when they become inmates of a private household. One great lady with a huge establishment in the country, where she had to employ on one occasion twenty-two private nurses, told us that her life during this time was a continual worry, owing to the absence of consideration which the majority of these nurses displayed. In consequence she became impressed with the feeling that character in a nurse was the most important of all qualifications, and she obtained her nurses on the next occasion from St. John the Divine, and found that her former troubles were at an end. What was the difference between the two types of nurses? The members of the sisterhood, had always a deep sense of duty as the mainspring of their action; the others were largely without it, being too much occupied with personal considerations which ought to be entirely absent when a nurse is on duty in charge of the sick. The unpopularity of certain trained nurses in private families is probably due to two causes. One has its origin in the public demand for young women who are not so suitable for private work as the older but thoroughly competent and more experienced trained nurse. We are confident that those who know most about nurses agree, that for private cases the elder women of the best type are undoubtedly the most suitable, and popular. Professor Osler's joke has been taken seriously by the unthinking public to an extent which is not creditable to the average intelligence of the men and women of our day and generation. In nursing, at any rate in private families, a good age should be an added qualification for the trained nurse. The other cause must be put down to the authorities of the hospital where the self-conscious nurse has been trained. If there be one place more than another in which every worker should be animated by the right spirit, and that spirit one which must tend to elevate all the workers, surely that place is a hospital for the relief of the sick and suffering.

This consideration raises another point which has recently given rise to some discussion in the press. Is it desirable that the day's duties in a hospital should commence with the assembling of all the staff so that all may join together in a few simple prayers? In the old days, where this practice prevailed, it was not uncommon for sectarianism to be introduced, by the action of some Roman Catholic priest, who forbade a member of the nursing staff to be present at such a service. The authorities did not raise the religious question; the form of prayers used was wholly unobjectionable; the rules properly provided that every member of the staff should commence the day's work by attending this simple early service, and the priest was clearly wrong in the attitude he assumed. This view was supported by the higher authorities in the Church of Rome, and it is now recognised by the ablest and most successful administrators that it is desirable that this system should prevail in every well regulated hospital. The efficiency of the whole hospital and the happiness of every member of the staff are best secured by commencing the day's work with some simple service, for in this way a continuous presence of the right spirit in the work is assured. The evening service should certainly not be compulsory, for it can well be believed, that after twelve or thirteen hours, mostly spent on duty, a nurse may feel such attendance irksome, in which case it is calculated to do her more harm than good.

### Abdominal Surgery.

By Harold Burrows, M.B., F.R.C.S., Assistant Surgeon to the Seamen's Hospital, Greenwich, and to the Bolingbroke Hospital, Wandsworth Common.

(*Concluded from page* 43.)

### Wound Complications.

There are three complications of abdominal wounds. They may become infected by septic organisms, they may burst open, or the resultant scar may yield so as to allow the formation of a post-operative or ventral hernia.

If on account of the patient's temperature or for other reason, the wound be inspected and found to be septic, it will need to be dressed daily. Sometimes the edges of a septic wound gape widely, especially when there has been severe tympanites. In such a case it may be well to draw the edges of the wound together by means of strapping applied over the gauze with which the wound is dressed.

If a wound bursts open, owing to inefficient suturing or to excessive muscular effort on the part of the patient, he will feel sudden severe pain, which will be followed by shock. On examining the dressings the nurse will perhaps find that some blood has soaked through, and so will be led to discover the accident. The medical attendant must be summoned at once, for it will be necessary to return any escaping omentum or bowel into the abdomen and to suture the wound again. For this an anæsthetic will probably be required.

Ventral hernia is due to yielding of the scar left by an abdominal wound. It is not uncommon after septic wounds, such as are caused by opening and draining an appendix abscess. To lessen the likelihood of his getting a ventral hernia, the patient should not be allowed to throw a heavy strain on the scar until the latter has become strong enough to bear the strain. So the patient must not be permitted to get up and walk about too soon; and if there is thought to be a chance of his acquiring a hernia a sufficient abdominal belt should be worn for the ensuing six months or longer.

### Intestinal Fistulæ.

Either as the result of operation or of disease, an opening may exist between one of the hollow viscera and the surface of the abdomen. Examples of this are gastrostomy wounds, gastric fistulæ caused by ulcers of the stomach, enterostomy wounds, fæcal fistulæ following appendicitis, colostomy wounds, and cholecystostomy wounds. In all these a good deal of care is required to prevent the escaping liquids from producing severe inflammation of the skin. As might be expected, the material from a gastric fistula is the most apt to damage the skin, and unless precautions are taken a severe dermatitis of the abdomen may be caused within twenty-four hours of the formation of the fistula.

The treatment consists in keeping the skin scrupulously clean and protecting it from the intestinal contents by a coating of simple ointment. After colostomy the fear of dermatitis is not so great as with openings into the upper portions of intestines, as the less liquid contents of this part of the bowel are not very irritating.

There is one particular accident that may follow colostomy. This is prolapse of a portion of bowel through the wound. When this occurs, the prolapsed bowel should be gently replaced, and an opinion formed as to the reason of its escape. It may be that some scybala are present, in which case the bowel above the opening should be washed out with soap and water by means of a catheter.

### Feeding.

Food may be introduced (1) by the mouth, (2) by the rectum, (3) subcutaneously, and (4) by inunction.

### Oral Feeding.

For practical purposes it may be taken that the stomach is incapable of digesting food for several hours after an abdominal operation; and even then the digestive power returns very slowly.

Nothing should be given by the mouth until the post-operation vomiting has ceased. When this cessation has occurred, the patient may be allowed small sips of hot boiled water; and if these do not cause nausea or sickness, feeding by the mouth may be commenced cautiously. But care must be taken to give food which can be absorbed with the least amount of effort.

Raw milk is one of the worst possible foods for these cases. It is teeming with bacteria, it forms solid curds in the stomach, and therefore is difficult to digest, and it does not stimulate peristalsis, so that constipation and tympanites result. Excepting the first, boiled milk also has these drawbacks. The following are suitable for administration after abdominal section :—Albumen water, dextrose solution, lactose solution, raisin tea, raw beef juice, thin Benger's food. Gelatine, plasmon, and other similar preparations may be useful also.

*Albumen water* is made by beating up the whites of three eggs in a pint of cold boiled water, and adding a little salt or lemon.

*Dextrose and lactose* may be given in a 10 per cent. solution in water.

*Raisin tea* is made by pouring water on to half its bulk of chopped raisins, stewing this for two hours, and then straining.

*Raw beef juice* must be prepared only from perfectly fresh meat. The beef juice should be mixed with some ordinary beef tea or meat extract, and should be suitably flavoured.

### Rectal Feeding.

Often it is impossible or undesirable to give a patient a full supply of nourishment by the mouth, and consequently rectal feeding must be used. Half an hour or an hour before giving a nutrient enema, the bowel should be washed out with warm water. In the actual administration of the nutrient enema, particular care must be taken not to introduce the fluid too quickly, or the rectum will rebel and refuse to retain the injection. The best method is to let the fluid run in from a glass funnel attached to a soft rectal tube, the funnel being held no higher than one foot above the rectum. If given slowly and carefully in this manner, as much as one pint may be retained by an adult. Albumen water with a teaspoonful of salt to the pint, or with 5 per cent. dextrose added makes the best enema, for it is unirritating and is easily absorbed.

### Subcutaneous Feeding.

The technique of this is precisely the same as that for subcutaneous injection of saline solution. The fluid used consists of 5 per cent. dextrose in normal saline solution, and must be boiled before injection. Subcutaneous feeding is of value when attempts at rectal feeding prove failures or are insufficient, or when it is expedient to administer as much nourishment as possible with the least delay.

### Inunction.

In infants and young children fat can be introduced through the skin by inunction. The method is rarely employed in abdominal cases, but it may be useful in congenital hypertrophic stenosis of the pylorus, where death from starvation is threatened. The skin of the back, chest, or thighs is washed with warm water, and some cod-liver oil or olive oil is rubbed in with the hand. The process may be repeated as often as necessary.

### The Nurses' Clinic.

### BURNS.

The treatment of burns, both local and constitutional, is of the greatest importance. In severe cases the constitutional is of more immediate importance than the application of local remedies, as the patient is suffering from "shock" in its very worst form, and should be treated accordingly. He should be wrapped in warm blankets, and if in a private house placed near the fire and some warm brandy and water given at once. In hospital, after the patient has been put in bed with plenty of warm clothing and hot bottles, most surgeons give a hypodermic injection of strychninæ, 1 to 5 minims according to the age of the patient, in addition to the stimulant already mentioned, and in very bad cases an intravenous injection of normal saline is usually given.

When the warmth of the patient has been restored, then attention may be directed to the seat of injury and the dressings applied to one limb at a time, taking care that the rest of the body is carefully covered up. In case of burns of the trunk, in children and in adults (if the conveniences are at hand), a warm boracic bath 100° F.), will ease the pain very much, as well as take off any charred remnants of the clothing. It is most important that the temperature of the bath be maintained as long as the patient is allowed to remain in it.

In superficial burns when there is only an inflamed condition of the skin, a saturated solution of bicarbonate of soda is very soothing, and can always be procured easily—lint or gauze soaked in this solution and covered with oiled silk makes a very good dressing. A solution of picric acid is also used with very good results as an application to burns or scalds. The solution is made by dissolving a drachm and a half of picric acid in three ounces of alcohol and two pints of distilled water, strips of sterilised gauze soaked in the lotion and covered with a layer of absorbent wool is applied, and kept on with a bandage for a couple of days, when the dressings should be carefully soaked off with the lotion and renewed.

When vesicles have been produced they should be snipped with a pair of sharp pointed scissors and the serum gently pressed out; at this stage the best dressing is, perhaps, some antiseptic greasy application such as boric acid ointment or eucalyptus ointment. They have the advantage of not sticking to the skin, and hence the dressings are not so painful.

If the true skin is burnt and destroyed it separates by ulceration. This burnt surface should be thoroughly disinfected to prevent profuse suppuration. If suppuration takes place and the tissues become inflamed, boric fomentations should be applied every four hours until the inflammation subsides and the discharge is diminished.

In most hospitals it is usual for the house surgeon to administer chloroform a time or two in cases of very extensive burns while the nurse is dressing the patient. The one object of the nurse should be to keep the wound clean and to prevent suppuration. The point is to try and promote rapid healing.

### Incidents in a Nurse's Life.

### SMALL-POX PATIENTS IN TENTS.

I was attached to a large nursing home in the North of England during a recent epidemic of small-pox.

"The medical officer of health requires a nurse for small-pox," rang out the telephone one day while I was waiting for a "case."

I expressed my willingness to go, so, receiving directions from the matron as to requirements for isolation probably for some time, I prepared to depart, and shortly after was being rapidly driven to my "case," the cab stopping at the town offices to take up the sanitary inspector, who accompanied me to my destination, three miles out of town.

With some curiosity I gazed out of the cab window to get a view of the situation of my temporary home.

### Where I had to Nurse.

The cab turned up a lonely lane, on one side of which lay a wood, and then in at a wide wooden gate.

"Here we are, nurse," said the sanitary inspector in a cheery voice as he jumped down and opened the door. "A bit lonely up here, but you will get used to that." Imagine a large piece of waste ground, all mud and puddles (for it was raining heavily), entirely open on three sides except for a low hedge and a few trees, and without even a wall to give a feeling of security. With sinking spirits I looked around. On this ground was pitched a large canvas tent, oblong in shape, with a wooden floor, round which ran hot-air pipes, the heat supplied by a coke stove on the outside of the tent. Inside the tent had been placed all the furniture and utensils which were likely to be required by the hospital and staff.

There were three low bedsteads erected, and on these were bundles of bedding mattresses, all waiting to be made up.

On the floor was a large tin bath full of pots, pans, and dishes—also a small paraffin stove and two lamps. One or two small wooden tables, a couple of chairs, and some wash basins completed the list.

### INCIDENTS IN A NURSE'S LIFE—*continued.*

### I SET TO WORK.

However, I did not stand very long gazing—my patient had not yet arrived—so after taking off my bonnet and cloak, which I did not put on again for many weeks, I turned round and tried to get things a bit ship-shape. First, there was the expected patient's bed to make up; then, it being impossible to air sheets and blankets without a fire, I hunted up two hot-water bottles out of the bath and a tin kettle. Next I tried to light the stove and was still busy with it when the inspector walked in, and seeing my difficulty proceeded to explain the working of the stove.

"You will want some water bringing, nurse," he said; "I will go and wake up that lazy scoundrel Rafferty and make him bring some."

Saying which he disappeared with the water-can. On returning he told me he had been at the back of the tent trying to make the coke stove burn in order to heat the pipes.

"That ass Rafferty ought to have had the tent at 65° by this time; instead the pipes are quite cold. I found the stove all choked with coal tar and have had such a bother with it."

### MY SOLE FACTOTUM.

He then proceeded to explain to me that "that ass Rafferty" was to be my sole factotum—to go up and down to the village on errands, keep the stove going, and bring the water, which was obtained from a spring some fields off.

"And where does this man sleep?" I inquired. "Oh, he goes home at present; what further arrangements may be made requires to be seen—there is no accommodation on the grounds."

"Besides this tent there is only a small zinc-covered hut where the food is kept, but so long as you have only one or two patients you will manage all right; if more come we shall have to get another tent. I must be off now, nurse—will look round again in a day or two. If you require anything in the meantime send a message by Rafferty," saying which the inspector walked away, and I was left alone except for Rafferty, who could be seen making his way across the fields with the water.

### ARRIVAL OF MY FIRST PATIENT.

I managed to get the tent and bed nice and warm before the patient arrived, which he did an hour later with his mother, and proved to be a boy of five years old. After giving her a cup of tea, the boy falling asleep, she quietly left us.

As the boy still slept I went to the tent door to look round. Oh! what a dismal prospect. Rain, rain, rain! and mud, mud, mud!

Rafferty was sitting at the door of the "cook-house"—as we afterward christened the little zinc hut—and seeing me came to inquire if I "wanted anything to-night."

"No, thank you," I replied.

"Then I will go home; but first let me show you how to close the tent."

Soon after this I was entirely alone in a field with a sick child of five years old.

I do not think I am naturally timid—nurses need to be strong-nerved—but I must say I felt a little that night like Robinson Crusoe might have felt on his desert island.

After waiting up some hours, hoping the doctor would come, I gave it up at last, and after closing the tent lay down near the boy.

### THE FIRST NIGHT.

I could not sleep, however. The flapping canvas and swaying lamps—which threatened to explode with every breath of wind—the feeling that I was "on duty" though no watching was required, all combined to keep me awake until early morning, when I dozed off.

Waking up about 6 A.M., I found my little patient sitting up in bed regarding me with great solemn eyes.

I rose, dressed quickly, opened the tent, and proceeded to prepare breakfast and make the child comfortable.

"Bobby" was very good and amused himself with the toys I had found until the afternoon, when the inevitable "howling," which every nurse who has had to do with children dreads, commenced in real earnest. Dear me! how that child did scream.

I tried everything to soothe him—played with him, carried him about in my arms—but the moment I laid him down he started again, crying for "Mammy Hinney, I wants you." "Mammy Hinney!" he kept screaming. He certainly did stop to eat his dinner and tea, but started again when finished. Towards evening, tired out, he fell asleep.

### THE DOCTOR'S VISIT.

"Well, nurse," he said, after a glance at the boy, "he's all right, but there is another case coming in to-day—a very bad one. I will be in to-morrow to see him. Rafferty tells me his people object to him going home at night; so he will have to sleep on the grounds."

"Doctor!" I exclaimed, "there is no place but the little zinc hut where we keep the food."

"I am afraid he will have to go in there, nurse. I will send up another bed for him."

"Doctor," I said, a little nervously, "will you kindly tell him not to come near the tent at all? I would much rather do my own sweeping—I would, in fact, rather be without the man if it were possible."

"I am afraid it is not possible, nurse; you must have a messenger at hand; but I will warn him to keep away from the tent; if you have any trouble just send a note to me."

"Yes, doctor," I replied doubtfully; for I felt that Rafferty could not be relied upon to take a note in such a case.

### WHISKY AS A DISINFECTANT.

The doctor proved himself a good friend to us during the ensuing trying weeks; but he made one great mistake that day when he gave the Irishman permission to take whisky to prevent infection.

Ever after that when we wanted Rafferty for anything we would find him lying dead drunk on his bed in the cook-house. We had to get rid of him at last, but that was not for some weeks later. After the doctor's departure he came along to say he was sleeping on the grounds that night—was then going to the village for provisions and whisky. Could he bring me any wine? On replying that I did not take anything of the sort, he went on his errands to the village, leaving me again alone.

### THE WORST PATIENT.

And now came the most trying time I had during the sixteen weeks of isolation.

A boy of fifteen years, suffering from small-pox in one of its worst forms, had also developed double pneumonia, and being very ill indeed was brought to the tent. That night and for many nights I sat up with him, giving him brandy, milk, and beef-tea in spoonfuls every few minutes, and trying to keep him warm with hot bottles. Rafferty had let the stove go out, reducing the temperature of the tent to 44°. I tried the paraffin stove inside, but it made so much smoke I put it out again.

The doctor came again the next day and examined the boy. Mentioning to him about the stove going out, he looked at it, explaining there was something defective in it, and said : "Sometimes when the wind is in the wrong direction it will not burn."

Rafferty overheard the words, and ever after, when the pipes were cold : "The wind was in the wrong direction!"

The doctor did not come again for two or three days, and I saw no one but Rafferty, except the boy's mother, who brought some clean linen.

The boy's temperature kept up; at night he was very restless and delirious. Rafferty annoyed me very much at this time, offering to watch by the patient while I slept. Of course this was out of the question.

Another time it was : "Nurse, open the tent about 4 A.M. and I will bring you a cup of tea." "No, thank you," I replied; "I can make tea inside on the stove." Again it was the offer for wine renewed and the insolent leer with which he accompanied the remark, "that it would be all right, and I ought to have it to keep up my strength."

### Feeling the Strain.

At such times I would walk away in disgust and wish I had another nurse for company. When fine I would cook the food at the tent door on a gipsy fire and eat my dinner there when possible, for a small-pox tent is not the most tempting dining-room. There were days when it rained so heavily it made it impossible to stir from the tent. Meanwhile Bobby had fits of screaming for "Mammy Hinney," but as he became convalescent these were fewer.

The boy Mike was no better. I was beginning to feel the strain of day and night nursing combined when one night he seemed rather worse than usual. I had looked for the doctor all that day, but he had not come, so I was obliged to send a message that night. The rain had poured all day and Rafferty had allowed the pipes to get cold and the temperature was again 44°. Fancy a patient with pneumonia and the ward at 44°.

### Arrival of the Sanitary Inspector.

So I sent my note, but instead of the doctor the sanitary inspector came, bringing a lantern, it being too dark to see his way. I explained how things were, telling him that it was enough to kill the boy almost, the tent so cold. He then went round to the stove, but could not make it "go," it being really out of order then and for days after. The doctor coming next day, I asked for another nurse, as Mike was too ill to be left for a moment.

"Can you not put him some milk and brandy on the locker, nurse, and let him get it himself while you lie down?" he said.

"He will take nothing, doctor, unless I spoon it down his throat; and then he is delirious at night, and I am afraid of him getting out of bed," I answered.

"Well, I will ring up another nurse to-morrow if he is no better," replied the doctor.

### More Patients and Another Nurse.

It was not until some days after this, however, that the doctor walked in one morning saying more patients were coming and a nurse—also a bell tent to sleep in—to take turns on night duty. How thankful I was to hear this!

Things changed after this. We had twelve more patients, men and women; another tent was erected for the females and a small one for the nurses. What an unspeakable comfort it was to be able to eat our food away from the sick ones, and to be able to sleep with both eyes shut! I had been there fourteen days and had scarcely any sleep, and was feeling the want of it; for, notwithstanding "Sister Dora," I have never yet met a nurse who could go without sleep for more than twenty-four hours and not feel the effect of it.

Most of these new cases were mild ones and able to get up pretty soon. Mike's two brothers and Bobby's mother and father were in. Mike continued ill for many weeks, but ultimately recovered. When the weather changed we brought him into the sunshine, and with night and day attention we persevered with the nourishment and brandy till he was out of danger. The boy himself was grateful, but his two brothers, rough mining men, gave us no end of trouble, making us very glad when they were discharged.

### A Capital Cook.

Of course we had all the cooking to do for our large family, and in this capacity our new nurse proved invaluable : she could prepare the daintiest dishes with the ease of a professional cook. No wonder the patients soon began to mend.

After the women patients were discharged we decided to do the washing ourselves, and so lessen the danger of infection by bringing outsiders to assist. At last all these first inmates were discharged after making a splendid recovery.

### New Patients.

We were preparing to close the "hospital," preparatory to going into "quarantine," when about two nights before leaving the doctor came to say we must fetch another case from the Industrial School. I went in a cab and carried the boy downstairs myself, for no one would go near him and he was too ill to stand. A few days after the other nurse had a similar journey for his brother—an epidemic of small-pox having broken out in the school. Severe cases both, but no complications arising, they made good recoveries.

### A Fearful Storm.

Before this, however, a terrible storm arose one night, commencing about 11 P.M., growing in strength until the cooking utensils rolled about the floor, making a dreadful din. The women's tent (then empty) was blown down, our bell tent following, leaving nurse and myself hanging to the ropes of the large tent, while our "man" tried in a futile way to drive in the loosening staves. Finding we were only wasting our time and strength, we went inside the tent and tried to prop it up with empty beds and ward furniture, bringing the patients into the middle of the tent for safety. This went on until about 4 A.M., when we found the tent was no longer safe and likely to collapse any moment. We then wrapped the patients up in blankets and carried them to the cook-house, where the man's bed came in very handy. The wind was still strong in the morning, but assistance being obtained, the bell tent was fixed. Meanwhile we placed the patients in our own beds, and were glad to find that they suffered no harm from their change of quarters.

### Leaving the Small-pox Camp.

A few weeks after this all the patients were discharged, and we were eagerly looking forward to a week in quarantine at the seaside.

Leaving everything nice and clean from a sanitary point of view, we drove away from the place, after spending sixteen not unpleasant weeks. As the cab turned into the lane the other nurse looking back broke into a laugh : "I am thinking," she said, "of those people who, thinking it was a circus, came to ask when the show would commence, and how quickly they fell back at the magic words 'small-pox.'"

"Yes," I replied, thoughtfully, "it was the most comforting thought I had during my first lonely fortnight. A small-pox hospital is not in much danger of molestation, however exposed and solitary may be the situation."

We re-entered the nursing home finally, deeply grateful to our visiting doctor, who kept us supplied with good things sent from his own resources, and who saw we wanted for nothing which he could obtain for us.

### Midwives' Total Abstinence League.

MEETING AT ST. PAUL'S CHAPTER HOUSE.

The second annual meeting of the Certified Midwives' Total Abstinence League was held, by permission of the Dean of St. Paul's, at the Chapter House on Friday last, at 3.30. A goodly contingent of midwives assembled somewhat earlier, and were conducted into the crypt and through the galleries of the Cathedral.

The Chapter House, with its panelled walls hung round with paintings of the great Cathedral, formed a fine setting to the gathering of midwives, mostly in uniform, who completely crowded out the long room. There must have been between two and three hundred present, and it was pleasant to notice that the old school had sent representatives, who were seated side by side with the smart, neatly-uniformed young members of the new one.

Dr. Annie M'Call took the chair, and remarked that since the inauguration of the League a year ago a large amount of quiet, steady temperance work had been done by both members and associates. They wanted by this meeting to try and stimulate the workers to endeavour to get other people to join this League, which had been started so that the certified midwives of England should have a temperance league of their own of good standing. She hoped that they would come to consider that they had not done their duty until they had joined the League. There was absolutely nothing scientific to be said against total abstinence. She hoped that those who took alcohol for pleasure would soon step out of the ranks of midwives. In their work they had to make the very best use of their powers of mind and body, and this was impossible so long as they took alcoholic drinks. She pleaded with them also on behalf of their patients. The present company would wield an untold influence over an enormous number of young mothers and little children. No one could do a better work than those who directly influenced the mothers of England. She added that she had herself been a total abstainer for thirty-one years.

Miss Le Geyt, who said that she had been a total abstainer for forty-five years, congratulated the assemblage on the fact that the grand work of midwifery was beginning to receive the recognition it deserved, and it was now considered necessary for the human race that midwives should be trained and certified. The motto of the League was "Strong to save." They had it in their power to do the utmost good in the world, but in order to do so it was absolutely necessary that they should all become total abstainers. The time of greatest temptation to women was when they were nursing or awaiting their time of trial; then they were tempted to take stout or beer. Their other motto, "It is not the will of our Father in heaven that one of these little ones shall perish," furnished them with a second reason. Alcohol increased the quantity but deteriorated the quality of the mother's milk. Were they going to help in any possible way to put the taint of alcoholism into poor little babies? Were those who assisted at the beginnings of life going also to assist at the beginnings of corruption? If they wished to influence a patient who wanted to take a glass of stout they must not grudge their trouble in trying to persuade her to substitute milk. Another reason for teetotalism was the saving it involved. A pint of beer a day was £3 10s. a year; in five years, with interest, this would be over £16, and in ten years over £34; and if they married it would be enough to furnish a house. Or they might gain from this saving enough to purchase an annuity and so provide for old age. The force of example was very strong, and she urged them to take the pledge for themselves and for their sisters. They had before them golden opportunities for good work. After the earthquake at San Francisco all the public-houses were closed, and in consequence the hospitals were closed, too, for want of patients. Miss Le Geyt then asked the total abstainers present to rise, when the greater number of those present at once stood up.

Miss May Hughes, daughter of the author of *Tom Brown's Schooldays,* urged the claims of total abstinence in moving terms.

Miss Alice Gregory, who has worked as a district midwife in the country for many years, and founded the Home for Mothers and Babies and Training of Midwives at Wood Street, Woolwich, opened in May last year, then said a few words. She said that she would rather be a midwife than anything else in the world. It was hard to realise what an army of babies the present company had brought into the world during the past year. A midwife met an extraordinary number of people, and must exert some sort of influence. It was very difficult to keep their work up to the standard of training. Two-thirds of the cases of blindness in asylums might be saved by midwives. It was difficult to overestimate their influence on their patients, who would be the better or the worse according to the character of the midwife. A midwife who showed that she could do her day's work on temperance drinks could do good work for the temperance cause.

Dr. Rock told the meeting that the League now numbered 160. For the sake of those who did not understand the terms of the League she explained that a member must have two qualifications: she must be a total abstainer and a certified midwife. Honorary members paid one shilling a year. Probationary members joining only for a short period. Associates were those who wished to join with the midwives in taking this pledge, and could be of either sex and of any occupation. It was hoped that the midwives would try and enlist their patients as associates.

Miss Ritchie, secretary of the Clapham Maternity Hospital, proposed a vote of thanks to the Dean of St. Paul's and the speakers, and this was seconded by Miss Paget, who, as a member of the Central Midwives Board, said that she had the sad duty of sitting on the Penal Board, and trying cases of midwives accused of being drunk while at a case.

Tea was then served in the hall, and before the company separated twenty-seven more midwives joined the League.

### Everybody's Opinion.

**[Correspondence on all subjects is invited, but we cannot in any way be responsible for the opinions expressed by our correspondents. No communication can be entertained if the name and address of the correspondent are not given as a guarantee of good faith, but not necessarily for publication. All correspondents should write on one side of the paper only.]**

NURSES' HOSTEL.

"Friend" writes:—I have lived at the Nurses' Hostel for seven years, and find it a most useful and comfortable home between my cases. I am, therefore, most anxious that the officials who are being unfairly maligned by unreasonable nurses shall feel that there are very many who appreciate their unfailing courtesy and consideration for our welfare. The hostel is intended for nurses who are actively engaged in their profession, and we, who have plenty of work, value it highly; and the spirit of discontent and rebellion to every form of law and order is stirred up and fostered by those who have little else to do than gossip and find fault. I trust that strangers who do not know the hostel and cannot speak from experience will come and prove for themselves that it is all I claim for it. We are excellently well fed, the house is beautifully clean, and the servants are exceptionally attentive, and what more can be expected for the very reasonable charges made?

"One of the Many who are Satisfied" writes:—As a tenant in the Nurses' Hostel for more than four years, I wish to enter my protest against the unjust statements that are being published against the excellent management that started the hostel, and has successfully brought it to its present state of usefulness and popularity, of which there is convincing proof in the constant demand for accommodation within its homely precincts. I should like to know where nurses can find such a home as is here offered them at the modest figure they are asked to pay. I think it will be found that the nurses who are in constant work have no complaints to make, but speak in the kindest and most grateful terms of their homes in Francis Street. As few restrictions as possible—where the nurses are drawn from all classes—are enforced, as can be easily seen by sending to the secretary for a copy of the rules, and terms which are inclusive. The hostel was primarily intended for nurses who have to earn their living by their work, and not for those who possess substantial supplementary incomes and therefore have large and extravagant ideas. Consequently every effort has been made, and that most successfully, to meet the requirements of the real workers. There is no obligation for any nurses who do not appreciate the hostel's arrangements to stay there against their will and pose as martyrs; there are more luxurious quarters to be found in London if they are willing to pay dearer for them; but my opinion as one who knows the hostel well is that nurses may go further afield and fare very much worse.

"A Nine Years' Resident" writes:—I should like the nurses to know that this is not the first time that this matter respecting the management of the nurses' business affairs at the hostel has been brought before the notice of the directors. Some years ago I wrote to the secretary stating how keenly many of the nurses, including myself, were feeling in regard to the indifference paid to our business affairs and asking for necessary reforms, some of which, I am pleased to hear, have now been granted. The managing director informed me that my letter was impertinent. The secretary threw it into the fire. Another director said that I had not a leg to stand upon—I have two. The result of the committee meeting was that I received a letter from the secretary stating that the nurses' business affairs would be carried on in future as heretofore. I felt this treatment very keenly, and took a copy of the letter that had given risen to them to a well-known solicitor to ask his opinion on it. He said that "the letter could by no means be called impertinent; that it was quite a proper businesslike letter and should be treated in a businesslike manner; and that the secretary should be made to aplogise for such unbusinesslike conduct." I let the matter drop, being single-handed, and gave up trying to work on my own account, and joined a co-operation, the lady superintendent of which refused—and I think rightly—to give doctors' instructions to anyone but the nurse who was to carry them out. The result was that I had to pay the hostel 3d. for letting me know I was wanted by my co-operation, 2d. to the public telephone, and, of course, 2d. the co-operation end, making in all 7d. for each message. Any complaint made to Miss Wood was usually met with the remark, "If you don't like it you can go," and I did go, as soon as I could make it convenient. I am merely writing this in order to show what impossible people the authorities of the hostel have been in dealing with matters that did not concern their own personal interest. Great credit is due to them for the financial success of the hostel company, but it is a pity that they have entirely ignored the fact that for the most part the success has been attained through the aid of those whom they have treated with so much discourtesy and, I may say, injustice.

### "SPIRITUAL EYE-SALVE."

"A Lover of Justice and Hater of Cant" writes: The "spiritual eye-salve" which "A Lover of Truth" prescribes, must be a special "directors' own" preparation, made on the premises, as after use one is to see injustice, offensive manners, and bullying, as "sound spiritual judgment," "self-denial," and "devotion to good works"! The latest attempt to whitewash the conduct of the managing and other directors of the Nurses' Hostel under the guise of religion makes one genuinely disgusted. I thank you for your straight and temperate article in this week's issue, and wish Miss Hulme success.

### THE NEED FOR THE REGISTRATION OF NURSING HOMES.

"One who has Suffered" writes: This is a subject which seems to have been forgotten of late though a number of letters appeared in The Hospital in 1905 on the above, and the "need" is still keenly felt. Before I begin, I may say that I am neither a young nor inexperienced nurse, and have had a very trying experience. Having worked for years in hospitals, principally in my old training school, I thought I should like to earn a little more money, in fact I was obliged to try to do so. I saw a very grand advertisement, emanating from a home, requiring all sorts of qualifications and thought myself lucky when I was accepted, although I was certificated and experienced. You can imagine my horror when I arrived at a tiny house which I had some difficulty in discovering, and when I went inside I found four other nurses who had been there some time waiting for work, and hardly room to turn round. I remained patient for three weeks, but at the end of that time I began to be very uneasy, and timidly asked the lady who ran the establishment if there was any chance of work. She coolly replied: "Nurses have no right to join co-ops. except they have money and patience." I need not add that I left very shortly and others followed my example, and I was glad afterwards, as I found out that the place was merely a venture and that the matron (as she called herself) had only had a few private cases in the town. We had to pay for board and lodging in advance, so we were much poorer for our experience, though ever so much wiser. There is another danger to be avoided, though I cannot speak from experience this time. It is with regard to "homes" who employ both salaried and co-op. nurses. I have been told, and believe it to be the truth, that those receiving a salary are sent out again and again while the poor co-op. nurses wait and pay for board, etc. In conclusion, I advise all to find out if possible everything about the institute they are about to join and if possible have an interview instead of being deceived by advertisement and sweet letters. as I was.

"F. B." writes: After being six years with the Norfolk Association, I engaged with a matron in the West of England as midwife at her maternity home. I soon found that I had made a mistake, and that objectionable persons came to the home. The matron was out every night till 11.30, leaving her children and the home to the care of a servant, and since I left she has been telling everyone I drank, but I have a paper signed by all my patients saying that I gave every satisfaction, which I have forwarded to the Central Midwives Board. I hope that a Bill for the registration of these homes will soon pass.

### WHAT IS A TRAINED NURSE?

"Marie" writes:—The "Breach of Contract" case brings up the question, What is a trained nurse? Evidently the judge at Brompton County Court considers the small amount of training Nurse Jukes possesses to be sufficient for her to be counted as such. On no other grounds could he have given judgment in her favour. The printed form sent out by the matron of St. Clement's Maternity Home distinctly states that only "fully trained" nurses are taken for a three months' training for the Central Midwives Board examination. Pupils without previous training are not prepared for Central Midwives Board examination under six months. A partially trained nurse is sometimes taken for a four months' course of training. Pupils are, of course, taken for shorter courses, but not for Central Midwives Board examination. During the period I was assistant matron at St. Clement's—three and a half years—I had considerable experience with midwifery pupils, and I am fully convinced that the work and training required to pass the examination of the Central Midwives Board cannot be thoroughly done in a shorter period. I wish that all the maternity training schools would be united on this point, and then we should have better midwives.

### SO-CALLED TRAINED NURSES' ASSOCIATIONS.

"A Trained Nurse" writes: With reference to "M. H.'s" letter in your issue of October 13 regarding the above associations, and a plea for central organisation in a former issue, I trust that the subject will be taken up by interested gentlewomen and physicians, and that a genuine "Trained Nurses' Association" will be started, separate from the many lay employments which at present seem to be mixed up with the numerous so-called "Trained Nurses' Associations," where a trained nurse could go at any time with confidence that the association was working for her benefit and those that employ her, instead of for their own "interests" in getting as much money out of her as possible before even attempting to procure her an appointment. Recently a friend of mine answered one of these associations' advertisements for "Trained Nurses—immediate engagement." The rules were 5s. booking fee, 1s. for stamps, and 2s. out of every pound earned in the year; and the immediate engagement was a "Temporary Puerperal Mental Case"—pay at the rate of £24 a year. My friend took the case for a fortnight and earned £1, out of which she had to pay the association 8s. I appeal to all who are interested in nursing to say if such a state of affairs could not be improved upon? This is only one out of many similar cases which I have come across.

[In the instance mentioned the nurse appears to have accepted the conditions offered. The Nurses' Co-operation, 8 New Cavendish Street, is such an institution as "A Trained Nurse" seems to desire. These institutions can only be formed with the mutual co-operation of a number of nurses.—Ed. The Hospital.

### NURSE'S RECREATIONS.

"A Hospital Sister" writes: The other evening I was feeling tired and weary, and I was so thankful that it was my evening off. I always find change of work is far greater rest than just sitting down and doing nothing. I started off for a walk, and on the way met a friend. She asked how I was, and when I replied, "Dead beat," she added, "Now, you just come along with me; I am going to join the Central Institute Swimming Club at Davis Street." I love swimming, so began to brighten up. When I got to the baths and heard the peals of laughter I began to laugh myself. Anything like the delight of that swim up and down that bath I can never describe. Surely there is nothing like the water for taking all the starch out of you and making you free and happy and at peace with your surroundings. I went back to my work quite a new creature. The things that seemed so impossible yesterday were quite easy. I felt free to minister to my patients as never before now. I am hoping to learn to dive. I wish that more members of my profession would try my tonic.

### A NARROW ESCAPE.

"A Hospital Nurse" writes: May I be permitted to make a few comments on "An Incident of Hospital Life," given in your issue of October 20? A probationer is described as going on night duty in a hospital for the first time, with two bad cases in the ward, one being an amputation of leg recently returned from the operating theatre. The nurse, we are told, is overwhelmed with the responsibility of her post, which a visit from the house surgeon only tends to increase, as he impresses her with the necessity of watching the amputated stump carefully, for profuse bleeding was likely to cause death; and having given instructions for the appliance of the tourniquet if necessary is leaving the ward, when the night nurse "calls after him" (I use the words of the writer of the incident), "Would he send her another nurse as she will require one?" To this the amiable house surgeon replied "that he would do as she 'wished.'" No mention is made of any other supervision during the night; so we naturally conclude that the house surgeon's last visit does service for a night sister, and that it rests entirely with the poor inexperienced little probationer to decide as to whether her duties demand assistance or not. A "candle" is used periodically by the nurse for the examination of the stump—and here let me add that not even in cottage hospitals have I heard of so primitive or dangerous a practice. Towards morning, the tired and anxious night nurse makes a final examination, and in doing so, "some inflammable material" placed "underneath the patient" manages to ignite, and not, I think, unnaturally the nurse seizes the "inflammable material" and stamps out the flame. This act is spoken of by the writer in terms of warmest praise and appreciation. Possibly the nurse's promptness was deserving of mention; but at the same time, the recital of this incident is more suggestive to me of the fond parent or admiring friend than of anyone initiated in the custom and etiquette of a hospital training."

### Appointments.

Barnsley Hall Asylum, Bromsgrove.—Miss S. A. Grice has been appointed matron. She was trained at Guy's Hospital, and has been sister at Graylingwell Hospital, West Sussex County Asylum; assistant matron St. Edmundsbury Private Asylum, Dublin; assistant matron at the Salop and Montgomery Counties Asylum, Shrewsbury; and second matron at the Lancashire County Asylum, Warrington. She has also had experience in private and district nursing, and holds the medico-psychological certificate.

Beckett Hospital, Barnsley.—Miss Lucy E. Elford has been appointed sister. She was trained at the General Hospital, Stroud, and has since been staff nurse at the National Hospital for the Paralysed and Epileptic, Queen Square, Bloomsbury, London, and charge nurse at the Brook Fever Hospital, Woolwich.

Chelsea Infirmary.—Miss Rose Manning and Miss Sarah A. Ward have been appointed charge nurses. Miss Manning was trained at the Royal Infirmary, Derby, and has since been charge nurse at the Ilkeston Hospital. Miss Ward was trained at the Clayton Hospital, Wakefield, and has since been staff nurse at the Royal Victoria Hospital, Belfast.

General Hospital, Weston-super-Mare.—Miss Agnes E. Watson has been appointed sister. She was trained at the Rugby Hospital and Guy's Hospital, London. She has since been ward sister at Lambeth Infirmary, theatre sister at the Acland Home, Oxford, and night superintendent at the Royal Surrey County Hospital, Guildford.

Marland Hospital, Rochdale.—Miss Lily Fieldhouse has been appointed sister. She was trained at the General Infirmary, Bolton, and at the Sanatorium, Astley; she has since been charge nurse at Chelmsford Infirmary.

Richmond Union Workhouse, Surrey.—Miss Florence R. Whitren has been appointed charge nurse. She was trained at Shirley Warren Infirmary, Southampton, and has since been charge nurse at Chelmsford Infirmary.

St. Albans Hospital, Herts.—Miss Gertrude Davies has been appointed charge nurse. She was trained at Oldham General Infirmary, and has since been charge nurse at Loughborough General Hospital, where she did matron's holiday duty.

Surrey County Asylum, Brookwood.—Miss Maud L. Barnes has been appointed charge night nurse. She was trained at St. Saviour's Infirmary, East Dulwich, S.E.

West End Hospital for Nervous Diseases, Welbeck Street, London.—Miss Charlotte Sidgwick has been appointed assistant matron. She was trained at the Westminster Hospital, where she was afterwards masseuse and performed sister's duties; she has since been out-patient sister at the West End Hospital, London. She holds the certificate of the Incorporated Society of Trained Masseuses.

### Notes and Queries.

**REGULATIONS.**

**The Editor is always willing to answer in his column, without any fee, all reasonable questions, as soon as possible.**

**But the following rules must be carefully observed.**

1. **Every communication must be accompanied by the name and address of the writer.**
2. **The question must always bear upon nursing, directly or indirectly.**

**If an answer is required by letter a fee of half-a-crown must be enclosed with the note containing the inquiry.**

*Advice Gratis.*

(48) I should like to know the address of the doctor whom it was stated gives advice gratis to nurses?—*Maternity Nurse.*

It is not unusual for medical men to give advice gratuitously to nurses.

*Maternity Nurse.*

(49) Are maternity nurses employed at the nursing homes? *Wheatley.*

Yes; watch our advertisement columns.

*Mental Nurse.*

(50) Can a mental nurse claim time off duty in a private house?—*Scotch.*

It is very unwise not to arrange to be off duty at reasonable times when you enter into an engagement. It would certainly be right to ask for it, even after engagement, as no one can maintain their health without relaxation from such trying duties.

*Home for Cripple.*

(51) Can you tell me of a home for a crippled and imbecile boy of eight years?—*Subscriber.*

Perhaps the Western Counties Idiot Asylum, Starcross, Devon, might help you, or the Children's Home, Littleton, near Guildford.

*Wanted a Training School.*

(52) Is the Hackney Infirmary a training school? (2) Is a three years' certificate given? (3) What is the salary? (4) At what London Hospital can I be trained and receive a salary?—*Brighton.*

(1) Yes. (2) Yes. (3) The salary to commence with is £10 per annum. (4) You can train at St. Bartholomew's, London, or the London Homœopathic Hospital, and receive £8 the first year. Or St. George's Hospital and the Middlesex Hospital, first year £10 and £12 respectively.

*Consumption.*

(53) Can you advise where to send a young consumptive man for open-air treatment?—*Kent.*

If there is no sanatorium in the neighbourhood, write to the Secretary, Mount Vernon Hospital, Hampstead, N.W., or Winsley Sanatorium, near Simpley Stoke.

(54) Do you know where a young lady suffering from phthisis could be admitted free of charge?—*Beta.*

Try the Hospital for Consumption and Diseases of the Chest, Brompton, S.W. They have a Samaritan Fund. If unsuccessful, try the other hospitals for consumption.

*Peat Moss.*

(55) Where can I procure peat moss, or is there any other dressing cheaper and better for cancer pads?—*District Nurse.*

Write to any of the large stores. Peat moss is cheaper than other dressings.

*Consumption.*

(56) Please tell me where a young asylum nurse could go, as she is suffering from consumption. Could she expect any help from the institution she has worked in?—*An Anxious One.*

The authorities of the asylum should certainly help, as the nurse became ill in their employment. Make an application, and write again if you want more information.

*Queen's Nurse.*

(57) How can I become a Queen's Nurse?—*White Heather.*

Write to the General Superintendent, Queen Victoria's Jubilee Institute for Nurses, 120 Victoria Street, S.W.

*Midwives' Club.*

(58) Can you give me the address of the Midwives' Club? —*W.*

If you mean the Midwives' Institute, it is 12 Buckingham Street, Strand, W.C.

*Uniform.*

(59) I am only partly trained, but am continuing my training in a few weeks. Can I wear uniform in the interim?—*Economy.*

Certainly you can.

*Home for Aged Woman.*

(60) Can you help me to find a home for an old lady who can employ herself with needlework? A small weekly payment if necessary.—*Chelsea.*

Try the Female Friendly Society of Charterhouse Square, Homes for the Aged Poor (4s. weekly), 86 Penge Road, Annerley, S.E., and St. Peter's Home, Kilburn.

*Training.*

(61) Can you tell me of a general hospital where they train probationers at the age of 19?—*Chum.*

No general hospitals receive probationers so young.

*Employment.*

(62) Can you tell me of an institution where I can be supplied with work on paying a fee?—*A. M. H.*

If you are a fully-trained nurse you should try and join some nurses' home or co-operation. The Nurses' Co-operation, 8 New Cavendish Street, is an institution to which nurses can belong, and receive all their earnings less a small percentage.

*Idiot Asylum.*

(63) Can you tell me of a home for a poor child who is imbecile and partly paralysed?—*Alpha.*

The Earlswood Idiot Asylum, or write for advice from the National Association for Promoting the Welfare of the Feeble-minded, 73 Denison Road, Vauxhall Bridge Road, S.W.

*Maternity Nursing.*

(64) Can you tell me of a home which I could join, holding only a certificate for monthly nursing, and having some experience of private mental nursing?—*Derby.*

You can only hear of employment by advertising, I fear.

*Nurse-Child.*

(65) I am anxious to take a nurse-child of gentle birth. What premium should I ask?—*Plaistow.*

We do not answer inquiries on this subject.

*Anxious for Work.*

(66) I am a widow thirty years of age, and have had a good deal of experience in nursing, but no consecutive training. Can I enter a hospital as probationer without paying a premium?—*Sydney.*

Yes. Write for "How to Become a Nurse," price 2s. 4d., from the Scientific Press, 28 Southampton Street, Strand, select a suitable hospital, and write to the matron for particulars.

*Nursing in Oporto.*

(67) Can you tell me of any nursing home or institution in Oporto?

We know of no institutions there for nurses.

*Motherless Child.*

(69) Can you tell me of a home which would receive a motherless baby of four months' old for a small payment?—*Chiswick.*

Write to St. John Baptist Nursery Home, 28 Upper Tooting Road, S.W., or you will find a long list of orphanages in the "Englishwoman's Year-Book," price 2s. 6d., which can be obtained from the Scientific Press, 28 Southampton Street, Strand, W.C.

*Deaf and Dumb.*

(70) Can you tell me of a deaf and dumb institution where a little girl of ten years could be accepted free?—*Sutton.*

Write to the Deaf and Dumb Asylum, Margate, or the British Asylum for Deaf and Dumb Females, 5 Bloomsbury Square, W.C.

*Superfluous Hairs.*

(71) Can you tell me of a depilatory which would be permanent and yet harmless? Many are advertised, but knowledge is required for selection.—*Blackbird.*

Electrolysis is the only permanent method, and it is quite harmless in the hands of a skilful and experienced expert.

*Rheumatism.*

(72) Can you tell me what to do for muscular rheumatism in the arm?—*Sussex.*

Consult a medical man, and in the meanwhile there can be no harm in rubbing the arm gently for about ten minutes with chloroform lotion and Elliman's embrocation.

**Handbooks for Nurses.**

| | Post Free. |
| --- | --- |
| "A Handbook for Nurses." (Dr. J. K. Watson) ... | 5s. 4d. |
| "Nurses' Pronouncing Dictionary of Medical Terms" | 2s. 0d. |
| "Art of Massage." (Creighton Hale) ... ... ... | 6s. 0d. |
| "Surgical Bandaging and Dressings." (Johnson Smith.) ... ... ... ... ... ... ... | 2s. 0d. |
| "Hints on Tropical Fevers." (Sister Pollard.) ... | 1s. 8d. |

Of all booksellers or of The Scientific Press, Limited, 28 & 29 Southampton Street, Strand, London, W.C.

### New Books for Nurses.

A System of Surgical Nursing. By A. N. M'Gregor, M.D., F.F.P.S.G. (Glasgow : David Bryce and Son. Pp. 554. Price 9s. net.)

Few diversions are more delightful than to watch day by day the healthy growth of flowers, children, or human institutions. And so with pride and pleasure we note the evolution of the modern nurse. Within the span of a generation she has grown from a woman with little knowledge and no discipline into the best type of womanhood that the world possesses. It is true that there are reactionary persons who argue that a severe training transforms a woman into a machine. But this is a crude and false idea. Cultivated intelligence was never yet an enemy to gentleness, and knowledge is invariably the servant of devotion.

These remarks are quite by the way; but they are the first thoughts which come to the mind on taking up for review Dr. M'Gregor's "System of Surgical Nursing," for the huge mass of instruction which its 554 pages contains fills one with a kind of amazement. And the wonder is none the less because the reviewer is a surgeon in close touch with scientific movements.

Dr. M'Gregor has arranged his book into forty-two chapters and an appendix. Commencing with what may be termed general principles of surgery, including such subjcts as hæmorrhage, sepsis, and the healing of wounds, the author deals carefully and thoroughly with dressings, instruments, preparation for operation, anæsthetics, and post-operation treatment. Then follows a descriptive account of the pathology and treatment of the surgical maladies affecting the different regions of the body. The management and feeding of patients, the administration of hypodermic injections, enemata, douches, and so forth, are discussed in considerable detail. Massage and electrical treatment each occupies a chapter, while the final one contains an admirable account of the arrangement of nurses' duties. The appendix is occupied by tables of weights and measures, methods of sterilisation of surgical materials, and other miscellaneous subjects.

Throughout, the author's descriptions and directions are easy to follow; and he has just struck the right balance between theory and practice. The only fault we find is that there are no illustrations, consequently the book looks rather uninteresting at first, although that impression disappears on closer investigation. The price is perhaps rather high for the average individual nurse, but for purposes of reading and reference, the work should have a place in every nurse's library. The volume is dedicated to Mrs. Strong, matron of the Glasgow Royal Infirmary.

### Resignations.

King Edward VII.'s Sanatorium.—Miss Blanche Trew, R.R.C., has resigned the matronship of King Edward VII.'s Sanatorium, Midhurst, to which she was appointed at the opening of the institution.

Hospital for Infectious Diseases, Clifton, Brighouse.—The resignation of Miss Lillie Waddington, who has been matron of the Hospital for Infectious Diseases at Clifton, Brighouse, since it was opened eight years ago, took place last week, prior to her marriage. At the usual Board meeting a resolution to the following effect was unanimously carried : "That the resignation be accepted, and the Board place on record its high appreciation of the manner in which Miss Waddington has discharged the duties of her office during the period of her service with the Board; and that the Board desire to express their best wishes for her future welfare."

### For Reading to the Sick.

THE COMMUNION OF SAINTS.

Dear dead! they have become
  Like guardian angels to us;
And distant heaven, like home,
  Through them begins to woo us;
Love that was earthly, wings
  Its flight to holier places;
The dead are sacred things
  That multiply our graces.

They whom we loved on earth
  Attract us now to heaven :
Who shared our grief and mirth
  Back to us now are given.
  They move with noiseless foot
  Gravely and sweetly round us,
And their soft touch hath cut
  Full many a chain that bound us.

*F. W. Faber.*

Believe that those who are gone are nearer us than ever; and that if (as I surely believe) they do sorrow over the mishaps and misdeeds of those whom they leave behind, they do not sorrow in vain. Their sympathy is a further education for them, and a pledge, too, of help—I believe of final deliverance—for those on whom they look down in love.—*Charles Kingsley.*

After this I beheld, and lo, a great multitude, which no man could number, of all nations, and kindreds, and people, and tongues, stood before the throne, and before the Lamb. And now we come to the mention of that innumerable multitude who have already entered on the rest of paradise, and the waiting for their Lord. And in doing this we are surely declaring, in open act, our full accord with that inspiriting declaration of our creed, "I believe in the communion of saints." For herein we declare that it is not only the great saints with whom we claim a living fellowship; into whose inheritance of suffering, of deeds, and of prayers we have entered; but that there is a communion between all the true saints of Christ; that we claim kindred with all; and that we bless God for all departed this life in His faith and fear. So that we are brought to-day to this doctrine of the communion of saints : and a glorious doctrine it is; kindling within our hearts, if it please God the Holy Ghost so to work upon us, more earnest desires after humility, and watchfulness, and trust, and powers of active service. For whilst it is good for us to be continually set alone in things spiritual; whilst it is true that religion is to each one of us so personal a matter that there can be no soundness in it, unless we are, in the singleness of our own spiritual being, often thus alone with God; yet it is true also that He has placed us in a company —in a goodly company—of His children; that there are of His ordering many steps before us on the waste over which we have to pass; that though upon it for our special comfort, far beyond all other aids, are left the footprints of the Lord our Saviour, when as the Virgin born He traversed its dry and sandy places. Yet, besides this, our gracious God, lest our courage should fail, has set before us an unnumbered company of all ages and conditions. They were once tried by all our weaknesses, and beset by all our dangers, but they have held on to the end and won the rest for which we long.—*Bishop Wilberforce.*

